# The plant Polycomb repressive complex 1 (PRC1) existed in the ancestor of seed plants and has a complex duplication history

**DOI:** 10.1186/s12862-015-0319-z

**Published:** 2015-03-13

**Authors:** Lidija Berke, Berend Snel

**Affiliations:** Theoretical Biology and Bioinformatics, Department of Biology, Faculty of Science, Utrecht University, Padualaan 8, 3584 CH Utrecht, Netherlands

**Keywords:** LHP1, EMF1, Ring1a, Ring1b, Bmi1a, Bmi1b, Bmi1c, VRN1, Paralogous replacement

## Abstract

**Background:**

Polycomb repressive complex 1 (PRC1) is an essential protein complex for plant development. It catalyzes ubiquitination of histone H2A that is an important part of the transcription repression machinery. Absence of PRC1 subunits in *Arabidopsis thaliana* plants causes severe developmental defects. Many aspects of the plant PRC1 are elusive, including its origin and phylogenetic distribution.

**Results:**

We established the evolutionary history of the plant PRC1 subunits (LHP1, Ring1a-b, Bmi1a-c, EMF1, and VRN1), enabled by sensitive phylogenetic methods and newly sequenced plant genomes from previously unsampled taxonomic groups.

We showed that all PRC1 core subunits exist in gymnosperms, earlier than previously thought, and that VRN1 is a recent addition, found exclusively in eudicots. The retention of individual subunits in chlorophytes, mosses, lycophytes and monilophytes indicates that they can moonlight as part of other complexes or processes. Moreover, we showed that most PRC1 subunits underwent a complex, duplication-rich history that differs significantly between Brassicaceae and other eudicots.

**Conclusions:**

PRC1 existed in the last common ancestor of seed plants where it likely played an important regulatory role, aiding their radiation. The presence of LHP1, Ring1 and Bmi1 in mosses, lycophytes and monilophytes also suggests the presence of a primitive yet functional PRC1.

**Electronic supplementary material:**

The online version of this article (doi:10.1186/s12862-015-0319-z) contains supplementary material, which is available to authorized users.

## Background

Correct regulation of gene expression is crucial for survival, and therefore organisms evolved elaborate mechanisms to regulate transcription through repression [1]. One of these mechanisms involves ubiquitination of histone H2A (H2Aub), mediated by the Polycomb repressive complex 1 (PRC1) [2]. H2Aub ultimately leads to chromatin compaction [3] and is especially important during development by making critical genes inaccessible for transcription. PRC1 is recruited to its target loci by binding the histone modification H3K27me3, a product of PRC2, or by an alternative, PRC2-independent mechanisms that are not completely known [4].

PRC1 in *Drosophila melanogaster*, which is a model organism for chromatin research, consists of four core components: Polyhomeotic (Ph), Polycomb (Pc), dRing and Posterior sex combs (Psc) [2]. Mammals have multiple paralogs of the core components. In contrast, plants have been long thought to lack PRC1. The absence of PRC1 in plants seemed an obvious conclusion as an initial screen of *Arabidopsis thaliana* histone modifications failed to find a ubiquitinated H2A residue [5], a hallmark of PRC1, and because orthologs of crucial PRC1 subunits were initially not detected in *A. thaliana* or other plant genomes [6].

Gradually, the composition of the plant PRC1 was pieced together and PRC1 was shown to have a biological function. The plant PRC1 is currently thought to contain five subunits [7]. The ring finger proteins Ring1a-b and Bmi1a-c are orthologs of *D. melanogaster* dRING and Psc, respectively. They have ubiquitination activity [8-11] and interact with TERMINAL FLOWER 2/LIKE HETEROCHROMATIN PROTEIN 1 (TFL2/LHP1) [10,11] that functionally replaces the role of Pc in H3K27me3 binding [12]. The *D. melanogaster* PRC1 subunit Ph has no orthologs in plants. The remaining two PRC1 subunits are plant-specific proteins. EMBRYONIC FLOWER 1 (EMF1) is a poorly conserved protein with few conserved motifs and no annotated domains [13]. It interacts with Ring1a-b and Bmi1a-c and is an indispensible component for H2A ubiquitination activity [10,11]. VERNALIZATION 1 (VRN1), the second plant-specific protein, is involved in vernalization [14]. As depletion of VRN1 causes a phenotype that is similar to other PRC1 mutants, VRN1 was proposed to be the fifth subunit of the complex [15]. Its function however is unknown, and no interactions with other PRC1 subunits have been discovered to date. Accordingly, some authors do not consider it a core subunit of this complex [7].

Mutants in PRC1 subunits show severe and pleiotropic abnormalities. For example, tissues of Ring1a/Ring1b double mutants dedifferentiate into callus [10], and EMF1 mutants develop incomplete flowers immediately upon germination [13,16]. LHP1 is involved in vernalization [17,18], and LHP1 mutants are smaller, have small curled leaves and flower early [19,20]. PRC1 is thus important for maintaining cell identity and controlling developmental transitions.

In comparison to animals, PRC1 in plants is still enigmatic [21]. While some orthologs of animal subunits were found and there is evidence for interactions between certain subunits, the biochemical evidence for existence of a PRC1 complex in plants is still sparse. It also remains to be investigated how the PRC1 subunits evolved in plants. Due to its important role in development, the time of its emergence has been and will be used to derive implications for its function [22]. However, the current literature disagrees on when all PRC1 subunits emerged. While mosses were first identified as the earliest branching plants with LHP1 and Ring1 [23], more recently PRC1 was suggested to be much younger: both LHP1 and Ring1 were found only in angiosperms, and EMF1 and VRN1 only in eudicots [24]. In this paper we are able to resolve these discrepancies by using more sensitive phylogenetic methods. Moreover, the availability of many recently published genomes and transcriptomes from previously neglected taxonomic groups enabled us to expand the inquiry into the phylogenetic distribution of PRC1 subunits by including gymnosperms and monilophytes. Our results demonstrate that PRC1 subunits appear in more early diverging plants than previously thought: Ring1 orthologs in chlorophytes, LHP1 and Bmi1 orthologs in mosses, lycophytes and monilophytes, and EMF1 orthologs in gymnosperms. Thus, all core subunits of PRC1 were already present in the ancestor of seed plants. The putative interacting protein VRN1 was a eudicot-specific invention that emerged in a relatively recent gene duplication. Moreover, plant PRC1 subunits underwent several rounds of duplications. Surprisingly, we uncovered three so far unrecognized paralogs of EMF1 stemming from two duplications before the gymnosperm-angiosperm split. We also resolved the duplication-rich history of other PRC1 subunits that has important consequence for inferring function of orthologs in non-Brassicaceae species. Lastly, we point out conserved motifs in some of the proteins that might have functions related to PRC1 complex and are therefore candidates for experimental inquiry to elucidate plant-specific molecular biology of this important protein complex.

## Results

To unravel the history of the plant PRC1 complex, we performed sensitive similarity searches and subsequently inferred phylogenetic trees for its subunits. This allowed us to determine the time of duplications and losses as well as the time of inventions for plant-specific proteins. We used 55 plant genomes, covering all major plant groups (Additional file 1). Among others, we use genomes of seven chlorophytes, and a gymnosperm. Genomes of early diverging plants are especially important in order to determine the time of invention as PRC1 was suggested to be already established by the time of angiosperm divergence [23-25]. We also used the recently published genome of the basal angiosperm *Amborella trichopoda*, as well as 6 monocot and 25 eudicot genomes. In addition, animal, fungal and SAR (stramenopiles, alveolates, and rhizaria) genomes were used as outgroups for proteins that already existed at the last common ancestor of eukaryotes (LECA). To supplement the missing or underrepresented plant groups we added gymnosperm and monilophyte sequences from transcriptome projects whenever applicable. Due to recently finished genome and transcriptome sequencing projects, this paper is the first to also include monilophytes and gymnosperms to elucidate the evolutionary history of PRC1.

### LHP1 is present in all multicellular plant lineages

**Figure 1:**
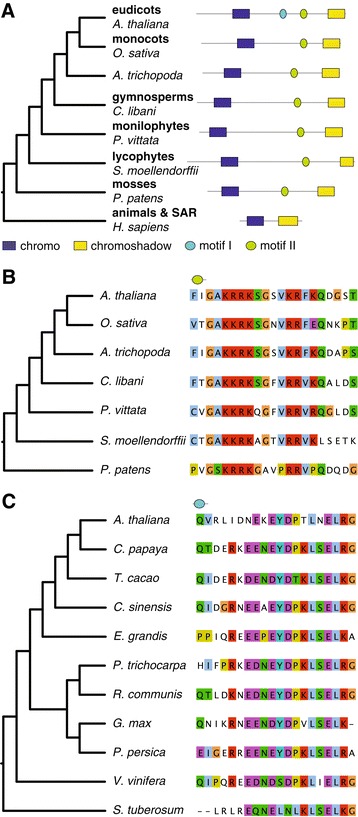
**LHP1 and its orthologs. (A)** A schematic gene tree of LHP1 orthologs (left panel) with domain structure from representative species in different groups (right panel). **(B)** Species tree and alignment of the plant-specific conserved motif. **(C)** Species tree and alignment of the eudicot-specific conserved motif.

LHP1 was first described as a plant homolog of *D. melanogaster* HETEROCHROMATIN PROTEIN 1 (HP1) [20]. While HP1 and its animal and fungal orthologs bind the histone modification H3K9me2 [26,27], LHP1 binds H3K27me3 [12]. We identified orthologs of LHP1 in animals, fungi and SAR (Figure 1A, Additional file 2), consistent with its presence in LECA. The plant LHP1 sequences cluster in a single orthologous group. The phylogenetic distribution of LHP1 includes all land plants (embryophytes). This is a much wider distribution than just the angiosperm group reported recently [24] and in agreement with the work of Hennig *et al.* [23]. All seven chlorophyte genomes lack LHP1 orthologs. This likely represents a secondary loss as our methods are sufficiently sensitive to easily recover orthologs in other eukaryotic supergroups. Using transcriptome data we show that LHP1 is also present in gymnosperms and monilophytes. In angiosperms, LHP1 is mostly a single-copy gene, and only genomes of species with relatively recent whole genome duplications (WGDs) often harbor several (2–4) LHP1 orthologs. This is also the case in Brassicaceae: there is one copy of LHP1 in the genomes of *A. thaliana*, *Arabidopsis lyrata*, *Capsella rubella* and *Thellungiella halophila*. The three copies in *Brassica rapa* suggest that the paralogs are derived from the *B. rapa*-specific whole-genome triplication.

LHP1 is characterized by a unique combination of an N-terminal chromo domain and a C-terminal chromoshadow domain [20]. The chromo domain in *A. thaliana* LHP1 binds H3K27me3 *in vivo* [12,28] whereas the chromoshadow domain [29] is involved in dimerization [20]. Plant LHP1 sequences contain an additional 200 amino acid region between chromo and chromoshadow domain compared to their animal orthologs (Figure 1B). The chromoshadow domain alone was shown to be sufficient for dimerization in *A. thaliana* [20] so this middle region likely functions in another process, perhaps mediating interactions with other proteins. Next to several low-complexity sections it also contains two conserved motifs (Figure 1). All plant LHP1 orthologs contain the second motif (Figure 1B), and the first (EYDPTLNELRG) is clearly present only in eudicots (Figure 1C). A shorter part of the second motif (RRKSGSV) corresponds to a potential substrate for PKA-type AGC kinase according to the Eukaryotic Linear Motif database (ELM) [30]. However, there is no evidence that this motif is phosphorylated [31], and the putative phosphorylation site (the serine residue) is not conserved in lycophytes, monilophytes and mosses. As the conserved motif is longer than only the putative phosphorylation motif, it is very likely to have a different or additional function. For example, two different nuclear localization signals (NLS) also match the second motif, one of which was already described [20]. However since these NLSs also cover only a small part of the conserved motif, the function is likely to extend beyond a NLS or a phosphorylation motif.

### Ring1a-b paralogs from pre-eudicot duplication were lost in the ancestor of Brassicaceae

**Figure 2:**
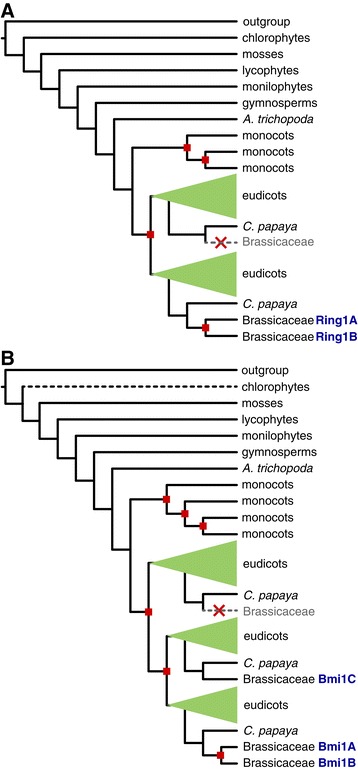
**Schematic gene trees of Ring1 (A) and Bmi1 (B).** Red squares show gene duplications. *A. thaliana* genes are denoted in blue letters. Brassicaceae are depicted in detail; inferred gene losses are shown with gray dashed line. Black dashed line: loss of one of the domains.

Ring1 and its orthologs in other eukaryotic supergroups consist of a ring finger domain, followed by a RAWUL domain. It originated before LECA [8]. In plants, the phylogenetic distribution of Ring1 proteins is somewhat controversial: they were found both in angiosperms and mosses [23] or described as angiosperm-specific [24]. The gene tree of Ring1 proteins (Figure 2A, Additional file 3) shows not only that that both moss and angiosperm genomes harbor Ring1 orthologs but also that already gymnosperm, monilophyte, lycophyte and chlorophyte genomes encode full-length Ring1 orthologs. *A. trichopoda*, the basal angiosperm, contains a single copy. Ring1 underwent two duplications in the monocot (or grass) ancestor, and subsequently underwent more species-specific duplications as well as losses.

Interestingly, the Ring1 gene was also duplicated in the ancestor of eudicots, resulting in two eudicot orthologous groups (Figure 2A). One of the two Ring1 copies was lost in Brassicaceae after the split from *Carica papaya*. To more precisely determine the time of loss, we added *Tarenaya hassleriana* orthologs to the gene tree [32] (Additional file 3). *T. hassleriana* belongs to Cleomaceae, a sister group to Brassicaceae. *T. hassleriana* sequences clustered in both eudicot orthologous groups. Thus the loss of Ring1 is Brassicaceae-specific. The second eudicot copy of Ring1 was subsequently duplicated in the α WGD [33], before the divergence of Brassicaceae. Therefore, the two paralogs in *A. thaliana*, Ring1a and Ring1b, originated relatively recently, and non-Brassicaceae eudicot lineages harbor a much more diverged pair of Ring1 proteins. This reciprocal duplication and loss in Brassicaceae could be an indication that Ring1a or Ring1b functionally replaces the lost Ring1 gene in the second orthologous group, an example of paralogous gene displacement [34].

### Bmi1a/b and Bmi1c duplicated in the ancestor of eudicots

Harboring the same domain structure as Ring1a-b proteins, Bmi1 proteins duplicated and diverged from Ring1 proteins already before LECA [8]. In plants, Bmi1 proteins are present in mosses, lycophytes, monilophyets and gymnosperms (Figure 2B, Additional file 4). Chlorophytes lost the RAWUL domain but retained the ring domain. Thus, the earliest plant lineage with full-length Bmi1 orthologs are indeed mosses [24].

The genome of *A. trichopoda* harbors a single Bmi1 gene, and three duplications in monocots ultimately yielded four monocot Ring1 orthologous groups. Two duplications in eudicots resulted in three eudicot orthologous groups. One contains Bmi1a and Bmi1b; these two paralogs emerged in the α WGD, a Brassicaceae-specific duplication [33]. Bmi1c, however, is a member of the second eudicot orthologous group; the divergence between Bmi1a/b and Bmi1c therefore predates eudicot speciation. The third eudicot orthologous group does not contain any Brassicaceae sequences, suggesting a loss after divergence from *C. papaya*. Therefore, similar to the Ring1 tree, the Brassicaceae-specific duplications and losses resulted in altered relationships between paralogs compared to other eudicots. Three Bmi1 gene copies in *Vitis vinifera*, for example, are not 1:1 orthologs to the three Bmi1 copies in *A. thaliana*, and their functions likely differ. One of the two recently duplicated paralogs (Bmi1a or Bmi1b) might functionally replace the lost paralog from the third eudicot orthologous group, the second example of paralogous gene displacement.

### EMF1 originated before gymnosperms

**Figure 3:**
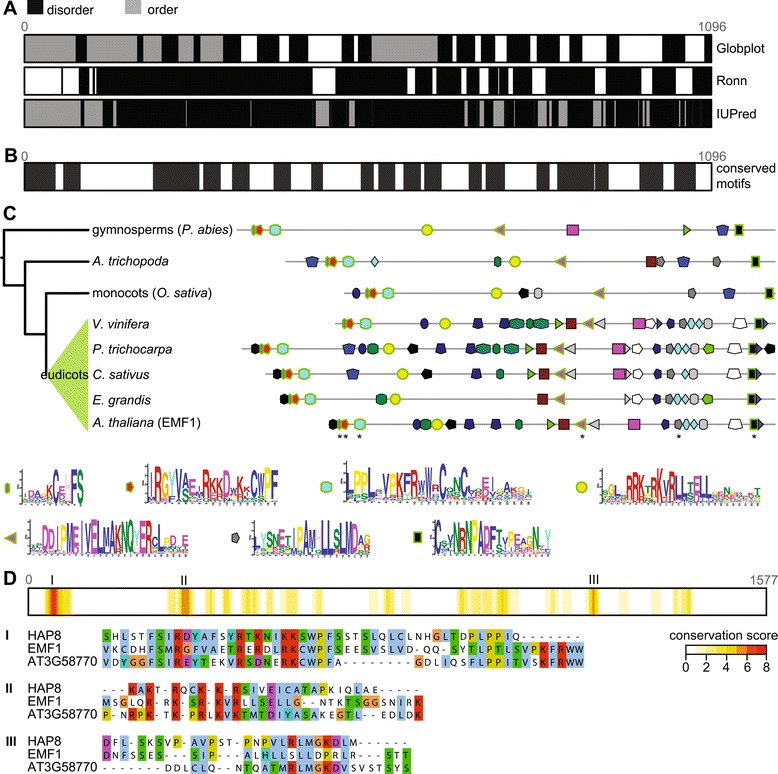
**EMF1 with its paralogs and orthologs. (A)** Disorder prediction by three different programs. **(B)** Conserved motifs predicted by meme, aligned to the disorder predictions. **(C)** Schematic gene tree of EMF1 and conserved motifs when comparing proteins in EMF1 orthologous group. Motifs that are conserved in all taxonomic groups are outlined in green. Stars at EMF1 mark motifs that have been previously described (the first two motifs were described as a single motif). The fifth already described motif (grey pentagon) is not present in gymnosperms. Nonsyntenic sporadic motifs that were likely false positives were removed for clarity of the figure. **(D)** Alignment conservation of three representative A. thaliana sequences, extracted from alignment of sequences of all three orthologous groups. As motifs do not take into account indels in sequences, this method returns different results.

EMF1 is a plant-specific protein with similar chemical properties to the C-terminal region of Psc, the *D. melanogaster* ortholog of Bmi1 [35], as well as a similar function: both interfere with transcription [25]. EMF1 has no annotated domains, and protein disorder prediction programs indicate that it is highly disordered (Figure 3A), with a small globular part at the N-terminal end that could in fact be a protein domain. Comparing disorder predictions to conserved motifs reveal that conserved motifs do not preferentially localize to either ordered or disordered regions (Figure 3B).

EMF1 was recently shown to be present in eudicots only [24] despite previous work showing its homologs in monocots [13,25]. Our homology searches and gene tree revealed that EMF1 originated earlier than previously thought: by using more sensitive methods we could recover EMF1 orthologs not only in monocots and eudicots but also in *A. trichopoda* and in gymnosperms (Figure 3C; Additional file 5). In monocots and eudicots, EMF1 orthologs are present mostly as single-copy genes. We could not recover EMF1 orthologs in mosses. However, sequence fragments in a monilophyte transcriptome [36] align with EMF1 alignment but are too short to be unambiguously identified as EMF1 orthologs. Monilophyte genome sequences are therefore needed to firmly establish the point of EMF1 invention.

Previous work showed that EMF1 has only few conserved motifs: nuclear localization signals, P-loop, and LXXLL elements [13] when compared to a putative rice ortholog. However with rice and lotus orthologs it shares five conserved motifs [25] that do not overlap with those found previously. By using the *de novo* motif search algorithm meme and more plant species than previously, we can show that the five conserved sections predicted by Calonje *et al.* [25] are indeed conserved in the entire EMF1 orthologous group (in Figure 3C shown as 6 motifs), from gymnosperms to eudicots. In gymnosperms, the fifth motif (Figure 3C, grey pentagon) is only weakly conserved and the motif search algorithm does not recover it. In addition, we find a new, well-conserved motif (Figure 3C, yellow circle) that corresponds to both a NLS and a phosphorylation site. Importantly, meme does not find gapped motifs and in case of insertions or deletions some motifs could have been missed. For example, the motif marked by a purple square is only a part of a longer motif with a highly conserved tryptophan and serine residue. However, as the introduction of gaps is necessary to compare the sequences, meme was able to find a shortened part in only few proteins.

Surprisingly, we found that the ancestral EMF1 gene duplicated twice in the ancestor of gymnosperms and angiosperms (Additional file 5). This resulted in three orthologous groups that, next to EMF1, contain three novel outparalogs. All four genes show a conserved N-terminal part that is followed by weakly conserved motifs (Figure 3D). The first orthologous group contains EMF1. The second orthologous group encompasses AT5G56240 and HAPLESS8 (HAP8) [37]; the two genes arose in a duplication before the speciation of Brassicaceae. The third orthologous group contains AT3G58770 that is annotated as unknown protein. Apart from HAP8, whose deletion hinders pollen tube growth, there is no functional data on the three outparalogs.

Except for lack of gymnosperm sequences in one of the orthologous groups (that is likely a result of secondary loss in gymnosperms), the phylogenetic distribution in each orthologous group covers all seed plants, including monocots, *A. trichopoda* and nearly all eudicot sequences. Monocot sequences tend to be truncated, however, and in the orthologous group with AT3G58770 several aligned loci are annotated as two separate genes.

### VRN1 is the youngest addition to PRC1

VRN1 was named after its role in vernalization; however, as its overexpression also causes a range of changes in plant organs, it seems to be also involved in more fundamental processes [14]. It does not interact with LHP1 [38] nor are there any reports of it interacting with other PRC1 subunits. While some suggest that VRN1 interacts with PRC1 only at a specific subset of target genes [7], others nevertheless assign it to PRC1 [15,38]. We include VRN1 in the overview for the purpose of completeness.

**Figure 4:**
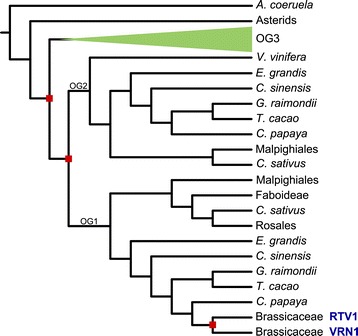
**Schematic gene tree of VRN1.** Red squares show gene duplications. Lineage-specific expansions are not depicted, with the exception of Brassicaceae. OG1-3 are the three orthologous groups as described in the main text. *A. thaliana* genes are denoted in blue letters.

VRN1 is characterized by two B3 domains that aspecifically bind DNA [14]. The B3 domain emerged in the plant lineage and occurs in a range of different domain combinations [39]. This promiscuity as well as numerous ancient and lineage-specific gene and domain duplications make it difficult to reconstruct the relation of VRN1 to other B3-domain containing proteins. The gene tree that we inferred shows that the evolutionary history of VRN1 is rich in duplications (Figure 4, Additional file 6). Two consecutive duplications after the split of asterids (*Solanum lycopersicum* and *Solanum tuberosum*) resulted in three orthologous groups. The VRN1 orthologous group has the shortest branches and therefore the slowest sequence evolution. Next to VRN1 it also contains RELATED TO VERNALIZATION 1 (RTV1) that lost its first B3 domain and only consists of a single B3 domain. The second orthologous group (OG2) underwent frequent gene losses. The third orthologous group (OG3) shows the longest branches. Lineage-specific duplications are frequent, and several genes consist of only a single B3 domain. This orthologous group also contains three *A. thaliana* outparalogs, AT1G49475, AT4G01580 and AT3G18960 (Additional file 6). None of them is functionally characterized.

Previous work found five VRN1 paralogs in *A. thaliana* and two in *P. trichocarpa* [24]. In contrast, we report only a single copy of VRN1 and a truncated paralog (RTV1), and indeed two *P. trichocarpa* orthologs. This result is probably due to phylogenetic trees enabling a more accurate way to distinguish inparalogs from outparalogs, i.e. to distinguish the VRN1 paralogs pre-dating ancient duplication events from those appearing afterwards.

## Discussion

**Figure 5:**
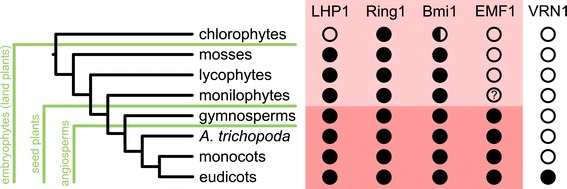
**Presence/absence of PRC1 components in the plant kingdom.** The red-shaded area represents the core PRC1 subunits. Bmi1 orthologs in chlorophytes have lost the RAWUL domain (half-circle). Monilophytes might have EMF1 orthologs but sequenced genomes are necessary to confirm this.

In this paper, we reconstruct the history and map the present day occurrences of PRC1 subunits. While the exact definition of PRC1 is under debate (reviewed in [21]), our inclusive definition of PRC1 encompassed the core subunits as well as VRN1. Most importantly, we show that the subunits are present in more early diverging species than previously thought. PRC1 subunits LHP1, Ring1 and Bmi1 were present in LECA. EMF1, a novel PRC1 subunit, originated in the ancestor of seed plants. PRC1 also gained novel interacting proteins, such as VRN1, which stems from a duplication in eudicots, and others [4]. Due to this as well as because of the losses in chlorophytes, PRC1 subunits show a sporadic distribution across early diverging plant species (Figure 5).

We are able resolve the discrepancy [23,24] regarding the distribution of PRC1 subunits across species due to improved phylogenetic methods and the availability of previously unsampled genomes and transcriptomes: in contrast to a recent study that reported limited phylogenetic distributions [24], all PRC1 subunits could be reliably inferred to have the same or even wider distributions than postulated earlier. Even though transcriptome data have some intrinsic drawbacks, e.g. dependence on the sampled tissues and gene expression levels, the analyzed data was sufficient to broadly estimate the phylogenetic distribution of the PRC1 subunits. The only exception is EMF1 for which, due to its very low sequence conservation, transcriptome data is inconclusive, and a sequenced monilophyte genome could help to firmly establish the time of invention. We expect that more accurate genomes and gene annotations, especially of species at key positions on the phylogenetic trees, will resolve the few remaining questions.

We cannot recover LHP1 in chlorophytes, in agreement with Hennig *et al.* [23]; mosses are the earliest LHP1-harboring plants in our dataset. The function of LHP1 might be particularly important in more complex, multicellular lineages and the loss of LHP1 might not have been detrimental for plants with simpler morphology. Genomes of chloropyhtes contain Ring1 orthologs and the ring domain of Bmi1. The absence of other PRC1 subunits strongly suggests that the ubiquitination activity of these proteins is important in other biological processes and that the proteins can moonlight as part of other protein complexes [40,41]. For example, PRC1 and PRC2 subunits were already shown to participate in other protein complexes in animals [42]. Psc, *D. melanogaster* ortholog of Bmi1, is also involved in ubiquitination of CYC-B as part of cell cycle regulation, a cellular process unrelated to PRC1 [43]. Bmi1a and Bmi1b have indeed been suggested in *A. thaliana* to act as ubiquitinases of protein DREB2A, a drought-inducible transcription factor [44]. Moonlighting of proteins in diverse complexes or processes could also explain the stark difference in phenotypes that were observed for different mutants of PRC1 subunits.

Interestingly, the gene trees for Ring1 and Bmi1 proteins show that gene duplications in Brassicaceae are accompanied by losses of paralogs in sister orthologous groups. These parallel events point to paralogous gene displacement. However, functional analysis of *A. thaliana* and non-Brassicaceae orthologs are necessarily to confirm this.

In summary, we revealed that the complete core PRC1 (consisting of LHP1, Ring1, Bmi1 and EMF1) existed in the ancestor of seed plants (Figure 5) and could have been instrumental in in establishing the complex developmental program of this group. The sporadic occurrence of PRC1 subunits in chlorophytes and mosses strongly suggests moonlighting of the proteins, especially since EMF1 is required for H2A ubiquitination activity in *A. thaliana* [10]*.* On the other hand, LHP1, Ring1 and Bmi1 might already act as a primitive but functional PRC1 in mosses. Such a primitive PRC1 could fulfill two roles: LHP1-mediated targeting to H3K27me3-marked nucleosomes and the ubiquitination activity provided by the Ring proteins. H3K27me3 is catalyzed by PRC2 that, similar to PRC1, also already existed in the ancestor of embryophytes [23]. PRC2 subunits MULTICOPY SUPRESSOR OF IRA1 (MSI1) and EMBRYONIC FLOWER 2 (EMF2) also interact with LHP1 in *A. thaliana* [45]. Regardless of whether these protein-protein interactions are widely conserved, PRC2 and a primitive PRC1 could have formed a repressive mechanism for transcription already in the ancestor of embryophytes.

## Conclusion

By using sensitive sequence search methods and inferring phylogenetic trees we show that the core PRC1 existed in the last common ancestor of seed plants. The presence of LHP1, Ring1 and Bmi1 in mosses, lycophytes and monilophytes also suggests the presence of a primitive yet functional PRC1. In addition, we uncover novel paralogs of EMF1. It remains to be shown whether their functional role is related to PRC1. Lastly, the duplication-rich history of many PRC1 subunits shows the intricate past of this complex and the necessity to use phylogenetic approaches to resolve the evolutionary relationships between paralogs and orthologs.

## Methods

### Genomes

55 genomes were downloaded from either Phytozome v 9.1 [46], Ensembl [47], their respective genome project web sites or NCBI (Additional file 1). We obtained the representative model or, if that was impossible, selected the gene model with the longest transcript. To supplement the genomic data, transcriptomes were obtained from a publication [36], onekp (onekp.com) (search with blastp was limited to the phylogenetic group of interest, and top BLAST hits were added to the sequences to construct the tree), and CoGe [48] for *T. hassleriana* [32].

### Phylogenetic analyses

Sequence search was performed with blastp 2.2.25 [49] (with softmasking) by using *A. thaliana* sequences (AT5G17690.1 (LHP1), AT1G03770.2 for (Ring1B), AT2G30580.1 (Bmi1A), AT5G11530.1 (EMF1), AT3G18990.1 (VRN1)) as queries. Reliable hits were aligned using MAFFT v7.127b [50] (settings genafpair, maxiterate 1000). We further refined sequence search by using hmmer 3.0 (http://hmmer.org/) to create a hidden Markov model – an alignment profile for more sensitive sequence search. Profiles were iteratively improved. We corrected cases where we encountered clearly erroneous gene models with either Augustus [51], exonerate 2.2.0 [52] (model protein2genome) or genewise [53] (global mode, modeled splice site). We reconfirmed unexpected absences with tblastn on Phytozome. The list of sequences was manually curated and extremely short sequences were removed. EMF1 orthologs were predicted as two separate genes in several genomes. For the purpose of reconstructing the evolutionary history of the loci, the two sequences were concatenated. Alignments are provided as Additional files 7, 8, 9, 10 and 11.

Alignment columns consisting of more than 90% gaps in the alignments were removed with trimAl v1.4.rev14 [54] (however the new alignment was required to not be shorter than 20% of initial alignment length). Selected columns are listed in the Additional file 12. Phylogenetic trees were inferred with RAxML v 7.9.5 [55] (rapid bootstrap analysis and search for best­scoring ML tree in a single run, 100 bootstraps), with the amino acid replacement model as determined by ProtTest 3.3 [56] (which was JTT + I + G for all trees), and visualized with iTol [57]. Because of their size and complexity, gene trees are represented as interpretations in the form of schematic trees in the main manuscript.

### Functional and other data/methods

Phosphorylation data were obtained from PhosphAt 4.0 [31]. *De novo* motif search in EMF1 and other orthologous groups was performed with meme v 4.9.1 [58] (any number of motifs, minimum motif width 10 aa, maximum motif width 150 aa, minimum number of sites 20). Linear motifs in LHP1 and EMF1 were identified using the Eukaryotic Linear Motif database (ELM) [30]. Disorder prediction was performed by globplot2 [59], ronn [60] and IUPred [61]. Visualization of alignments and calculation of conservation scores was done with Jalview v 2.8 [62].

## Availability of supporting data

All supporting data is available as additional files.

## Additional files

Additional file 1:
**List of genomes.**
Additional file 2:
**LHP1 gene tree.**
Additional file 3:
**Ring1 gene tree.**
Additional file 4:
**Bmi1 gene tree.**
Additional file 5:
**EMF1 gene tree.**
Additional file 6:
**VRN1 gene tree.**
Additional file 7:
**LHP1 alignment.**
Additional file 8:
**Ring1 alignment.**
Additional file 9:
**Bmi1 alignment.**
Additional file 10:
**EMF1 alignment.**
Additional file 11:
**VRN1 alignment.**
Additional file 12:
**Alignment details.**

## Abbreviations

PRC1: Polycomb repressive complex 1
H2Aub: Ubiquitination of histone H2A
LHP1: LIKE HETEROCHROMATIN PROTEIN 1
EMF1: EMBRYONIC FLOWER 1
VRN1: VERNALIZATION 1
WGD: Whole genome duplication
OG: Orthologous group
NLS: Nuclear localization signal
LECA: Last eukaryotic common ancestor
