# Supplementary material for: The plant Polycomb repressive complex 1 (PRC1) existed in the ancestor of seed plants and has a complex duplication history
Source: BMC Evol Biol. 2015 Mar 13;15:44. doi: 10.1186/s12862-015-0319-z (PMC4397884; doi:10.1186/s12862-015-0319-z)
Supplement: Additional file 1: — List of genomes. [file 12862_2015_319_MOESM1_ESM.pdf]

Additional file 1. List of genomes used.

Species code	Species name	Generic name	Genome version	Source/ reference	Taxonomic group
AmbTri	*Amborella trichopoda*		v1.0, evm_run27	[1]	Angiosperm
AquCoe	*Aquilegia coerulea*	Colorado blue columbine	JGI 8X assembly v1.0, annotation v1.2a	[2]	Eudicot
AraLyr	*Arabidopsis lyrata*	Lyrate rockcress	JGI release v1.0	[2, 3]	Eudicot
AraTha	*Arabidopsis thaliana*	Thale cress	TAIR10	[2, 4]	Eudicot
BatPra	*Bathycoccus prasinos*		Release 15jul2011	[5]	Chlorophyte
BraDis	*Brachypodium distachyon*	Purple false brome	JGI 8x assembly release v1.0 of strain Bd21 with JGI/MIPS PASA annotation v1.2	[2, 6]	Monocot
BraRap	*Brassica rapa*	Turnip mustard	Annotation v1.2 on assembly v1.1	[2, 7]	Eudicot
CaeEle	*Caenorhabditis elegans*	Nematode	WBcel235	[8, 9]	Metazoan
CapRub	*Capsella rubella*	Red shepherd's purse	JGI annotation v1.0 on assembly v1	[2, 10]	Eudicot
CarPap	*Carica papaya*	Papaya	ASGPB release of 2007	[2, 11]	Eudicot
ChlVar	*Chlorella variabilis*		Annotation v1.0 on assembly v1.0	[12]	Chlorophyte
CitSin	*Citrus sinensis*	Sweet orange	JGI v1.1 annotation on v1 assembly	[2, 13]	Eudicot
ClaRei	*Chlamydomonas reinhardtii*		JGI v5.0 assembly, annotation v5.3.1	[2, 14]	Chlorophyte
CocSub	*Coccomyxa subellipsoidea*		JGI v2.0 assembly and annotation	[2, 15]	Chlorophyte
CucSat	*Cucumis sativus*	Cucumber	Roche 454-XLR assembly and JGI v1.0 annotation	[2]	Eudicot
CyaMer	*Cyanidioschyzon merolae*		http://merolae.biol.s.u-tokyo.ac.jp/download, downloaded on 16.09.2013	[16]	Red alga
CyaPar	*Cyanophora paradoxa*		http://cyanophora.rutgers.edu/cyanophora/, downloaded on 11.10.2013	[17]	Glaucophyte
DroMel	*Drosophila melanogaster*	Common fruit fly	Assebbly BDGP5 with annotation FlyBase release 5.46	[8, 18]	Metazoan
EucGra	*Eucalyptus grandis*	Rose gum, flooded gum	JGI assembly v1.0, annotation v1.1	[2, 19]	Eudicot
FraVes	*Fragaria vesca*	Woodland strawberry	v1.1 assembly, v1.0 annotation	[2, 20]	Eudicot
FusOxy	*Fusarium oxysporum*		FO2 2011-02-Ensembl Fungi	[8, 21]	Fungus

GalSul	*Galdieria sulphuraria*		PRJNA13023, GCA_000341285.1	[22], NCBI BioProject PRJNA13023	Red alga
GlyMax	*Glycine max*	Soybean	JGI Glyma1.1 annotation of the chromosome-based Glyma1 assembly	[2, 23]	Eudicot
GosRai	*Gossypium raimondii*	Cotton	JGI annotation v2.1 on assembly v2.0	[2, 24]	Eudicot
HomSap	*Homo sapiens*	Human	GRCh37.p12	[8]	Metazoan
LacBic	*Laccaria bicolor*		Assembly v2, annotation 20110203	[25]	Fungus
LinUsi	*Linum usitatissimum*	Flax	BGI v1.0 on assembly v1.0	[2, 26]	Eudicot
ManEsc	*Manihot esculenta*	Cassava	Assembly version 4, JGI annotation v4.1	[2, 27]	Eudicot
MedTru	*Medicago truncatula*	Barrel medic	Mt3.5v4 on assembly MedtrA17_3.5 from the Medicago Genome Sequence Consortium	[2, 28]	Eudicot
MicPus	*Micromonas pusilla*		RCC299; JGI v3.0 assembly and annotation	[2, 29]	Chlorophyte
MimGut	*Mimulus guttatus*	Monkey flower	V2.0	[2, 30]	Eudicot
NeuCra	*Neurospora crassa*		NC12	[31]	Fungus
OrySat	*Oryza sativa*	Rice	MSU Release 7.0 of the Rice Genome Annotation	[2, 32]	Monocot
OstLuc	*Ostreococcus lucimarinus*		JGI v2.0 assembly and annotation	[2, 33]	Chlorophyte
PanVir	*Panicum virgatum*	Switchgrass	JGI v0.0 annotation on assembly v0	[2]	Monocot
PhaVul	*Phaseolus vulgaris*	Common bean	JGI annotation v1.0 on assembly v1.0 using published ESTs, and JGI RNAseq	[2, 34]	Eudicot
PhyInf	*Phytophthora infestans*		T30-4	[35]	SAR
PhyPat	*Physcomitrella patens*		JGI assembly release v1.1 and COSMOSS annotation v1.6	[2, 36, 37]	Moss
PicAbi	*Picea abies*	Norway spruce	v1.0	[38]	Gymnosperm
PopTri	*Populus trichocarpa*	Black cottonwood, western poplar	JGI assembly release v3.0, annotation v3.0	[2, 39]	Eudicot
PruPer	*Prunus persica*	Peach	JGI release v1.0	[2, 40]	Eudicot
RicCom	*Ricinus communis*	Castor bean plant	TIGR release 0.1	[2, 41]	Eudicot
SacCer	*Saccharomyces cerevisiae*	Baker's yeast	S288C	[42, 43]	Fungus
SelMoe	*Selaginella moellendorffii*		JGI v1.0 assembly and annotation	[2, 44]	Lycophyte
SetIta	*Setaria italica*	Foxtail millet	JGI 8.3X chromosome-scale assembly v2.0,	[2, 45]	Monocot

			annotation v2.1		
SolLyc	*Solanum lycopersicum*	Tomato	SGNTomato Genome Project ITAG2.3	[2, 46]	Eudicot
SolTub	*Solanum tuberosum*	Potato	DM1-3 516R44 (CIP801092) Genome Annotation v3.4 mapped to pseudomolecule sequence	[2, 47]	Eudicot
SorBic	*Sorghum bicolor*	Sorghum	v2.1	[2, 48]	Monocot
TetThe	*Tetrahymena thermophila*		Oct2008	[49]	SAR
ThaPse	*Thalassiosira pseudonana*		PRJNA34119, GCA_000149405.2	[50]	SAR
TheCac	*Theobroma cacao*	Cocoa tree	D. Gilbert public gene set 8 Mar 2012 on assembly v1.1	[2, 51]	Eudicot
TheHal	*Thellungiella halophila / Eutrema salsugineum*	Salt cress	JGI annotation v1.0 on assembly v1	[2, 52]	Eudicot
VitVin	*Vitis vinifera*	Common grape vine	March 2010 12X assembly and annotation from Genoscope	[2, 53]	Eudicot
VolCar	*Volvox carteri*		JGI annotation 2.0 on assembly v2	[2, 54]	Chlorophyte
ZeaMay	*Zea mays*	Maize	5b.60 annotation (filtered set) of the maize "B73" genome v2 produced by the Maize Genome Project	[2, 55]	Monocot

References:

1. Amborella Genome Project: **The Amborella genome and the evolution of flowering plants.** *Science* 2013, **342**:1241089.

2. Goodstein DM, Shu S, Howson R, Neupane R, Hayes RD, Fazo J, Mitros T, Dirks W, Hellsten U, Putnam N, Rokhsar DS: **Phytozome: a comparative platform for green plant genomics.** *Nucleic Acids Res* 2012, **40**(Database issue):D1178–86.

3. Hu TT, Pattyn P, Bakker EG, Cao J, Cheng J-F, Clark RM, Fahlgren N, Fawcett JA, Grimwood J, Gundlach H, Haberer G, Hollister JD, Ossowski S, Ottilar RP, Salamov AA, Schneeberger K, Spannagl M, Wang X, Yang L, Nasrallah ME, Bergelson J, Carrington JC, Gaut BS, Schmutz J, Mayer KFX, Van de Peer Y, Grigoriev IV, Nordborg M, Weigel D, Guo Y-L: **The *Arabidopsis lyrata* genome sequence and the basis of rapid genome size change**. *Nat Genet* 2011, **43**:476–481.

4. Lamesch P, Berardini TZ, Li D, Swarbreck D, Wilks C, Sasidharan R, Muller R, Dreher K, Alexander DL, Garcia-Hernandez M, Karthikeyan AS, Lee CH, Nelson WD, Ploetz L, Singh S, Wensel A, Huala E: **The Arabidopsis Information Resource (TAIR): improved gene annotation and new tools.**

Nucleic Acids Res 2012, **40**(Database issue):D1202–10.

5. Moreau H, Verhelst B, Couloux A, Derelle E, Rombauts S, Grimsley N, Van Bel M, Poulain J, Katinka M, Hohmann-Marriott MF, Piganeau G, Rouzé P, Da Silva C, Wincker P, Van de Peer Y, Vandepoele K: **Gene functionalities and genome structure in *Bathycoccus prasinos* reflect cellular specializations at the base of the green lineage.** *Genome Biol* 2012, **13**:R74.

6. Vogel JP, Garvin DF, Mockler TC, Schmutz J, Rokhsar D, Bevan MW, Barry K, Lucas S, Harmon-Smith M, Lail K, Tice H, Schmutz Leader J, Grimwood J, McKenzie N, Bevan MW, Huo N, Gu YQ, Lazo GR, Anderson OD, Vogel Leader JP, You FM, Luo M-C, Dvorak J, Wright J, Febrer M, Bevan MW, Idziak D, Hasterok R, Garvin DF, Lindquist E, et al.: **Genome sequencing and analysis of the model grass *Brachypodium distachyon***. *Nature* 2010, **463**:763–768.

7. Wang X, Wang H, Wang J, Sun R, Wu J, Liu S, Bai Y, Mun J-H, Bancroft I, Cheng F, Huang S, Li X, Hua W, Wang J, Wang X, Freeling M, Pires JC, Paterson AH, Chalhoub B, Wang B, Hayward A, Sharpe AG, Park B-S, Weisshaar B, Liu B, Li B, Liu B, Tong C, Song C, Duran C, et al.: **The genome of the mesopolyploid crop species *Brassica rapa*.** *Nat Genet* 2011, **43**:1035–1039.

8. Flicek P, Amode MR, Barrell D, Beal K, Billis K, Brent S, Carvalho-Silva D, Clapham P, Coates G, Fitzgerald S, Gil L, Girón CG, Gordon L, Hourlier T, Hunt S, Johnson N, Juettemann T, Kähäri AK, Keenan S, Kulesha E, Martin FJ, Maurel T, McLaren WM, Murphy DN, Nag R, Overduin B, Pignatelli M, Pritchard B, Pritchard E, Riat HS, et al.: **Ensembl 2014.** *Nucleic Acids Res* 2014, **42**(Database issue):D749–55.

9. Harris TW, Baran J, Bieri T, Cabunoc A, Chan J, Chen WJ, Davis P, Done J, Grove C, Howe K, Kishore R, Lee R, Li Y, Muller H-M, Nakamura C, Ozersky P, Paulini M, Raciti D, Schindelman G, Tuli MA, Van Auken K, Wang D, Wang X, Williams G, Wong JD, Yook K, Schedl T, Hodgkin J, Berriman M, Kersey P, et al.: **WormBase 2014: new views of curated biology.** *Nucleic Acids Res* 2014, **42**(Database issue):D789–93.

10. Slotte T, Hazzouri KM, Ågren JA, Koenig D, Maumus F, Guo Y-L, Steige K, Platts AE, Escobar JS, Newman LK, Wang W, Mandáková T, Vello E, Smith LM, Henz SR, Steffen J, Takuno S, Brandvain Y, Coop G, Andolfatto P, Hu TT, Blanchette M, Clark RM, Quesneville H, Nordborg M, Gaut BS, Lysak MA, Jenkins J, Grimwood J, Chapman J, et al.: **The *Capsella rubella* genome and the genomic consequences of rapid mating system evolution.** *Nat Genet* 2013, **45**:831–835.

11. Ming R, Hou S, Feng Y, Yu Q, Dionne-Laporte A, Saw JH, Senin P, Wang W, Ly BV, Lewis KLT, Salzberg SL, Feng L, Jones MR, Skelton RL, Murray JE, Chen C, Qian W, Shen J, Du P, Eustice M, Tong E, Tang H, Lyons E, Paull RE, Michael TP, Wall K, Rice DW, Albert H, Wang M-L, Zhu YJ, et al.: **The draft genome of the transgenic tropical fruit tree papaya (*Carica papaya* Linnaeus).** *Nature* 2008, **452**:991–996.

12. Blanc G, Duncan G, Agarkova I, Borodovsky M, Gurnon J, Kuo A, Lindquist E, Lucas S, Pangilinan J, Polle J, Salamov A, Terry A, Yamada T, Dunigan DD, Grigoriev IV, Claverie J-M, Van Etten JL: **The *Chlorella variabilis* NC64A genome reveals adaptation to photosymbiosis, coevolution with viruses, and cryptic sex.** *Plant Cell* 2010, **22**:2943–2955.

13. Wu GA, Prochnik S, Jenkins J, Salse J, Hellsten U, Murat F, Perrier X, Ruiz M, Scalabrin S, Terol J, Takita MA, Labadie K, Poulain J, Couloux A, Jabbari K, Cattonaro F, Del Fabbro C, Pinosio S, Zuccolo A, Chapman J, Grimwood J, Tadeo FR, Estornell LH, Muñoz-Sanz JV, Ibanez V, Herrero-Ortega A, Aleza

P, Pérez-Pérez J, Ramón D, Brunel D, et al.: **Sequencing of diverse mandarin, pummelo and orange genomes reveals complex history of admixture during citrus domestication.** *Nat Biotechnol* 2014, **32**:656–662.

14. Merchant SS, Prochnik SE, Vallon O, Harris EH, Karpowicz SJ, Witman GB, Terry A, Salamov A, Fritz-Laylin LK, Maréchal-Drouard L, Marshall WF, Qu L-H, Nelson DR, Sanderfoot AA, Spalding MH, Kapitonov VV, Ren Q, Ferris P, Lindquist E, Shapiro H, Lucas SM, Grimwood J, Schmutz J, Cardol P, Cerutti H, Chanfreau G, Chen C-L, Cognat V, Croft MT, Dent R, et al.: **The *Chlamydomonas* genome reveals the evolution of key animal and plant functions.** *Science* 2007, **318**:245–250.

15. Blanc G, Agarkova I, Grimwood J, Kuo A, Brueggeman A, Dunigan DD, Gurnon J, Ladunga I, Lindquist E, Lucas S, Pangilinan J, Pröschold T, Salamov A, Schmutz J, Weeks D, Yamada T, Lomsadze A, Borodovsky M, Claverie J-M, Grigoriev IV, Van Etten JL: **The genome of the polar eukaryotic microalga *Coccomyxa subellipsoidea* reveals traits of cold adaptation.** *Genome Biol* 2012, **13**:R39.

16. Matsuzaki M, Misumi O, Shin-I T, Maruyama S, Takahara M, Miyagishima S-Y, Mori T, Nishida K, Yagisawa F, Nishida K, Yoshida Y, Nishimura Y, Nakao S, Kobayashi T, Momoyama Y, Higashiyama T, Minoda A, Sano M, Nomoto H, Oishi K, Hayashi H, Ohta F, Nishizaka S, Haga S, Miura S, Morishita T, Kabeya Y, Terasawa K, Suzuki Y, Ishii Y, et al.: **Genome sequence of the ultrasmall unicellular red alga *Cyanidioschyzon merolae* 10D.** *Nature* 2004, **428**:653–657.

17. Price DC, Chan CX, Yoon HS, Yang EC, Qiu H, Weber APM, Schwacke R, Gross J, Blouin NA, Lane C, Reyes-Prieto A, Durnford DG, Neilson JAD, Lang BF, Burger G, Steiner JM, Löffelhardt W, Meuser JE, Posewitz MC, Ball S, Arias MC, Henrissat B, Coutinho PM, Rensing SA, Symeonidi A, Doddapaneni H, Green BR, Rajah VD, Boore J, Bhattacharya D: ***Cyanophora paradoxa* genome elucidates origin of photosynthesis in algae and plants.** *Science* 2012, **335**:843–847.

18. Adams MD: **The Genome Sequence of *Drosophila melanogaster***. *Science* 2000, **287**:2185–2195.

19. Myburg AA, Grattapaglia D, Tuskan GA, Hellsten U, Hayes RD, Grimwood J, Jenkins J, Lindquist E, Tice H, Bauer D, Goodstein DM, Dubchak I, Poliakov A, Mizrachi E, Kullan ARK, Hussey SG, Pinard D, van der Merwe K, Singh P, van Jaarsveld I, Silva-Junior OB, Togawa RC, Pappas MR, Faria DA, Sansaloni CP, Petroli CD, Yang X, Ranjan P, Tschaplinski TJ, Ye C-Y, et al.: **The genome of *Eucalyptus grandis*.** *Nature* 2014, **510**:356–362.

20. Shulaev V, Sargent DJ, Crowhurst RN, Mockler TC, Folkerts O, Delcher AL, Jaiswal P, Mockaitis K, Liston A, Mane SP, Burns P, Davis TM, Slovin JP, Bassil N, Hellens RP, Evans C, Harkins T, Kodira C, Desany B, Crasta OR, Jensen RV, Allan AC, Michael TP, Setubal JC, Celton J-M, Rees DJG, Williams KP, Holt SH, Ruiz Rojas JJ, Chatterjee M, et al.: **The genome of woodland strawberry (*Fragaria vesca*).** *Nat Genet* 2011, **43**:109–116.

21. Ma L-J, van der Does HC, Borkovich KA, Coleman JJ, Daboussi M-J, Di Pietro A, Dufresne M, Freitag M, Grabherr M, Henrissat B, Houterman PM, Kang S, Shim W-B, Woloshuk C, Xie X, Xu J-R, Antoniw J, Baker SE, Bluhm BH, Breakspear A, Brown DW, Butchko RAE, Chapman S, Coulson R, Coutinho PM, Danchin EGJ, Diener A, Gale LR, Gardiner DM, Goff S, et al.: **Comparative genomics reveals mobile pathogenicity chromosomes in *Fusarium*.** *Nature* 2010, **464**:367–373.

22. Schönknecht G, Chen W-H, Ternes CM, Barbier GG, Shrestha RP, Stanke M, Bräutigam A, Baker BJ, Banfield JF, Garavito RM, Carr K, Wilkerson C,

Rensing SA, Gagneul D, Dickenson NE, Oesterhelt C, Lercher MJ, Weber APM: **Gene transfer from bacteria and archaea facilitated evolution of an extremophilic eukaryote.** *Science* 2013, **339**:1207–1210.

23. Schmutz J, Cannon SB, Schlueter J, Ma J, Mitros T, Nelson W, Hyten DL, Song Q, Thelen JJ, Cheng J, Xu D, Hellsten U, May GD, Yu Y, Sakurai T, Umezawa T, Bhattacharyya MK, Sandhu D, Valliyodan B, Lindquist E, Peto M, Grant D, Shu S, Goodstein D, Barry K, Futrell-Griggs M, Abernathy B, Du J, Tian Z, Zhu L, et al.: **Genome sequence of the palaeopolyploid soybean.** *Nature* 2010, **463**:178–183.

24. Paterson AH, Wendel JF, Gundlach H, Guo H, Jenkins J, Jin D, Llewellyn D, Showmaker KC, Shu S, Udall J, Yoo M-J, Byers R, Chen W, Doron-Faigenboim A, Duke MV, Gong L, Grimwood J, Grover C, Grupp K, Hu G, Lee T-H, Li J, Lin L, Liu T, Marler BS, Page JT, Roberts AW, Romanel E, Sanders WS, Szadkowski E, et al.: **Repeated polyploidization of *Gossypium* genomes and the evolution of spinnable cotton fibres.** *Nature* 2012, **492**:423–427.

25. Martin F, Aerts A, Ahrén D, Brun A, Danchin EGJ, Duchaussoy F, Gibon J, Kohler A, Lindquist E, Pereda V, Salamov A, Shapiro HJ, Wuyts J, Blaudez D, Buée M, Brokstein P, Canbäck B, Cohen D, Courty PE, Coutinho PM, Delaruelle C, Detter JC, Deveau A, DiFazio S, Duplessis S, Fraissinet-Tachet L, Lucic E, Frey-Klett P, Fourrey C, Feussner I, et al.: **The genome of *Laccaria bicolor* provides insights into mycorrhizal symbiosis.** *Nature* 2008, **452**:88–92.

26. Wang Z, Hobson N, Galindo L, Zhu S, Shi D, McDill J, Yang L, Hawkins S, Neutelings G, Datla R, Lambert G, Galbraith DW, Grassa CJ, Geraldes A, Cronk QC, Cullis C, Dash PK, Kumar PA, Cloutier S, Sharpe AG, Wong GK-S, Wang J, Deyholos MK: **The genome of flax (*Linum usitatissimum*) assembled de novo from short shotgun sequence reads.** *Plant J* 2012, **72**:461–473.

27. Prochnik S, Marri PR, Desany B, Rabinowicz PD, Kodira C, Mohiuddin M, Rodriguez F, Fauquet C, Tohme J, Harkins T, Rokhsar DS, Rounsley S: **The Cassava Genome: Current Progress, Future Directions.** *Trop Plant Biol* 2012, **5**:88–94.

28. Young ND, Debellé F, Oldroyd GED, Geurts R, Cannon SB, Udvardi MK, Benedito VA, Mayer KFX, Gouzy J, Schoof H, Van de Peer Y, Proost S, Cook DR, Meyers BC, Spannagl M, Cheung F, De Mita S, Krishnakumar V, Gundlach H, Zhou S, Mudge J, Bharti AK, Murray JD, Naoumkina MA, Rosen B, Silverstein KAT, Tang H, Rombauts S, Zhao PX, Zhou P, et al.: **The *Medicago* genome provides insight into the evolution of rhizobial symbioses.** *Nature* 2011, **480**:520–524.

29. Worden AZ, Lee J-H, Mock T, Rouzé P, Simmons MP, Aerts AL, Allen AE, Cuvelier ML, Derelle E, Everett MV, Foulon E, Grimwood J, Gundlach H, Henrissat B, Napoli C, McDonald SM, Parker MS, Rombauts S, Salamov A, Dassow Von P, Badger JH, Coutinho PM, Demir E, Dubchak I, Gentemann C, Eikrem W, Gready JE, John U, Lanier W, Lindquist EA, et al.: **Green evolution and dynamic adaptations revealed by genomes of the marine picoeukaryotes *Micromonas*.** *Science* 2009, **324**:268–272.

30. Hellsten U, Wright KM, Jenkins J, Shu S, Yuan Y, Wessler SR, Schmutz J, Willis JH, Rokhsar DS: **Fine-scale variation in meiotic recombination in *Mimulus* inferred from population shotgun sequencing.** *Proc Natl Acad Sci USA* 2013, **110**:19478–19482.

31. Galagan JE, Calvo SE, Borkovich KA, Selker EU, Read ND, Jaffe D, FitzHugh W, Ma L-J, Smirnov S, Purcell S, Rehman B, Elkins T, Engels R, Wang S,

Nielsen CB, Butler J, Endrizzi M, Qui D, Ianakiev P, Bell-Pedersen D, Nelson MA, Werner-Washburne M, Selitrennikoff CP, Kinsey JA, Braun EL, Zelter A, Schulte U, Kothe GO, Jedd G, Mewes W, et al.: **The genome sequence of the filamentous fungus *Neurospora crassa***. *Nature* 2003, **422**:859–868.

32. Ouyang S, Zhu W, Hamilton J, Lin H, Campbell M, Childs K, Thibaud-Nissen F, Malek RL, Lee Y, Zheng L, Orvis J, Haas B, Wortman J, Buell CR: **The TIGR Rice Genome Annotation Resource: improvements and new features.** *Nucleic Acids Res* 2007, **35**(Database issue):D883–7.

33. Palenik B, Grimwood J, Aerts A, Rouzé P, Salamov A, Putnam N, Dupont C, Jorgensen R, Derelle E, Rombauts S, Zhou K, Otillar R, Merchant SS, Podell S, Gaasterland T, Napoli C, Gendler K, Manuell A, Tai V, Vallon O, Piganeau G, Jancek S, Heijde M, Jabbari K, Bowler C, Lohr M, Robbens S, Werner G, Dubchak I, Pazour GJ, et al.: **The tiny eukaryote *Ostreococcus* provides genomic insights into the paradox of plankton speciation.** *Proc Natl Acad Sci USA* 2007, **104**:7705–7710.

34. Schmutz J, McClean PE, Mamidi S, Wu GA, Cannon SB, Grimwood J, Jenkins J, Shu S, Song Q, Chavarro C, Torres-Torres M, Geffroy V, Moghaddam SM, Gao D, Abernathy B, Barry K, Blair M, Brick MA, Chovatia M, Gepts P, Goodstein DM, Gonzales M, Hellsten U, Hyten DL, Jia G, Kelly JD, Kudrna D, Lee R, Richard MMS, Miklas PN, et al.: **A reference genome for common bean and genome-wide analysis of dual domestications.** *Nat Genet* 2014, **46**:707–713.

35. Haas BJ, Kamoun S, Zody MC, Jiang RHY, Handsaker RE, Cano LM, Grabherr M, Kodira CD, Raffaele S, Torto-Alalibo T, Bozkurt TO, Ah-Fong AMV, Alvarado L, Anderson VL, Armstrong MR, Avrova A, Baxter L, Beynon J, Boevink PC, Bollmann SR, Bos JIB, Bulone V, Cai G, Cakir C, Carrington JC, Chawner M, Conti L, Costanzo S, Ewan R, Fahlgren N, et al.: **Genome sequence and analysis of the Irish potato famine pathogen *Phytophthora infestans***. *Nature* 2009, **461**:393–398.

36. Rensing SA, Lang D, Zimmer AD, Terry A, Salamov A, Shapiro H, Nishiyama T, Perroud P-F, Lindquist EA, Kamisugi Y, Tanahashi T, Sakakibara K, Fujita T, Oishi K, Shin-I T, Kuroki Y, Toyoda A, Suzuki Y, Hashimoto S-I, Yamaguchi K, Sugano S, Kohara Y, Fujiyama A, Anterola A, Aoki S, Ashton N, Barbazuk WB, Barker E, Bennetzen JL, Blankenship R, et al.: **The *Physcomitrella* genome reveals evolutionary insights into the conquest of land by plants.** *Science* 2008, **319**:64–69.

37. Zimmer AD, Lang D, Buchta K, Rombauts S, Nishiyama T, Hasebe M, Van de Peer Y, Rensing SA, Reski R: **Reannotation and extended community resources for the genome of the non-seed plant *Physcomitrella patens* provide insights into the evolution of plant gene structures and functions.** *BMC Genomics* 2013, **14**:498.

38. Nystedt B, Street NR, Wetterbom A, Zuccolo A, Lin Y-C, Scofield DG, Vezzi F, Delhomme N, Giacomello S, Alexeyenko A, Vicedomini R, Sahlin K, Sherwood E, Elfstrand M, Gramzow L, Holmberg K, Hällman J, Keech O, Klasson L, Koriabine M, Kucukoglu M, Käller M, Luthman J, Lysholm F, Niittylä T, Olson A, Rilakovic N, Ritland C, Rosselló JA, Sena J, et al.: **The Norway spruce genome sequence and conifer genome evolution.** *Nature* 2013, **497**:579–584.

39. Tuskan GA, DiFazio S, Jansson S, Bohlmann J, Grigoriev I, Hellsten U, Putnam N, Ralph S, Rombauts S, Salamov A, Schein J, Sterck L, Aerts A, Bhalerao RR, Bhalerao RP, Blaudez D, Boerjan W, Brun A, Brunner A, Busov V, Campbell M, Carlson J, Chalot M, Chapman J, Chen GL, Cooper D, Coutinho PM, Couturier J, Covert S, Cronk Q, et al.: **The Genome of Black Cottonwood, *Populus trichocarpa* (Torr. & Gray)**. *Science* 2006,

313:1596–1604.

40. International Peach Genome Initiative, Verde I, Abbott AG, Scalabrin S, Jung S, Shu S, Marroni F, Zhebentyayeva T, Dettori MT, Grimwood J, Cattonaro F, Zuccolo A, Rossini L, Jenkins J, Vendramin E, Meisel LA, Decroocq V, Sosinski B, Prochnik S, Mitros T, Policriti A, Cipriani G, Dondini L, Ficklin S, Goodstein DM, Xuan P, Del Fabbro C, Aramini V, Copetti D, Gonzalez S, et al.: **The high-quality draft genome of peach (*Prunus persica*) identifies unique patterns of genetic diversity, domestication and genome evolution.** *Nat Genet* 2013, **45**:487–494.

41. Chan AP, Crabtree J, Zhao Q, Lorenzi H, Orvis J, Puiu D, Melake-Berhan A, Jones KM, Redman J, Chen G, Cahoon EB, Gedil M, Stanke M, Haas BJ, Wortman JR, Fraser-Liggett CM, Ravel J, Rabinowicz PD: **Draft genome sequence of the oilseed species *Ricinus communis*.** *Nat Biotechnol* 2010, **28**:951–956.

42. Cherry JM, Hong EL, Amundsen C, Balakrishnan R, Binkley G, Chan ET, Christie KR, Costanzo MC, Dwight SS, Engel SR, Fisk DG, Hirschman JE, Hitz BC, Karra K, Krieger CJ, Miyasato SR, Nash RS, Park J, Skrzypek MS, Simison M, Weng S, Wong ED: **Saccharomyces Genome Database: the genomics resource of budding yeast.** *Nucleic Acids Res* 2012, **40**(Database issue):D700–5.

43. Engel SR, Dietrich FS, Fisk DG, Binkley G, Balakrishnan R, Costanzo MC, Dwight SS, Hitz BC, Karra K, Nash RS, Weng S, Wong ED, Lloyd P, Skrzypek MS, Miyasato SR, Simison M, Cherry JM: **The reference genome sequence of *Saccharomyces cerevisiae*: then and now.** *G3 (Bethesda)* 2014, **4**:389–398.

44. Banks JA, Nishiyama T, Hasebe M, Bowman JL, Gribskov M, dePamphilis C, Albert VA, Aono N, Aoyama T, Ambrose BA, Ashton NW, Axtell MJ, Barker E, Barker MS, Bennetzen JL, Bonawitz ND, Chapple C, Cheng C, Correa LGG, Dacre M, DeBarry J, Dreyer I, Elias M, Engstrom EM, Estelle M, Feng L, Finet C, Floyd SK, Frommer WB, Fujita T, et al.: **The *Selaginella* Genome Identifies Genetic Changes Associated with the Evolution of Vascular Plants**. *Science* 2011, **332**:960–963.

45. Bennetzen JL, Schmutz J, Wang H, Percifield R, Hawkins J, Pontaroli AC, Estep M, Feng L, Vaughn JN, Grimwood J, Jenkins J, Barry K, Lindquist E, Hellsten U, Deshpande S, Wang X, Wu X, Mitros T, Triplett J, Yang X, Ye C-Y, Mauro-Herrera M, Wang L, Li P, Sharma M, Sharma R, Ronald PC, Panaud O, Kellogg EA, Brutnell TP, et al.: **Reference genome sequence of the model plant *Setaria*.** *Nat Biotechnol* 2012, **30**:555–561.

46. Tomato Genome Consortium: **The tomato genome sequence provides insights into fleshy fruit evolution.** *Nature* 2012, **485**:635–641.

47. Potato Genome Sequencing Consortium, Xu X, Pan S, Cheng S, Zhang B, Mu D, Ni P, Zhang G, Yang S, Li R, Wang J, Orjeda G, Guzman F, Torres M, Lozano R, Ponce O, Martinez D, la Cruz De G, Chakrabarti SK, Patil VU, Skryabin KG, Kuznetsov BB, Ravin NV, Kolganova TV, Beletsky AV, Mardanov AV, Di Genova A, Bolser DM, Martin DMA, Li G, et al.: **Genome sequence and analysis of the tuber crop potato.** *Nature* 2011, **475**:189–195.

48. Paterson AH, Bowers JE, Bruggmann R, Dubchak I, Grimwood J, Gundlach H, Haberer G, Hellsten U, Mitros T, Poliakov A, Schmutz J, Spannagl M, Tang H, Wang X, Wicker T, Bharti AK, Chapman J, Feltus FA, Gowik U, Grigoriev IV, Lyons E, Maher CA, Martis M, Narechania A, Otillar RP, Penning BW, Salamov AA, Wang Y, Zhang L, Carpita NC, et al.: **The *Sorghum bicolor* genome and the diversification of grasses.** *Nature* 2009, **457**:551–556.

49. Eisen JA, Coyne RS, Wu M, Wu D, Thiagarajan M, Wortman JR, Badger JH, Ren Q, Amedeo P, Jones KM, Tallon LJ, Delcher AL, Salzberg SL, Silva JC, Haas BJ, Majoros WH, Farzad M, Carlton JM, Smith RK, Garg J, Pearlman RE, Karrer KM, Sun L, Manning G, Elde NC, Turkewitz AP, Asai DJ, Wilkes DE, Wang Y, Cai H, et al.: **Macronuclear genome sequence of the ciliate *Tetrahymena thermophila*, a model eukaryote.** *PLoS Biol* 2006, **4**:e286.

50. Armbrust EV, Berges JA, Bowler C, Green BR, Martínez D, Putnam NH, Zhou S, Allen AE, Apt KE, Bechner M, Brzezinski MA, Chaal BK, Chiovitti A, Davis AK, Demarest MS, Detter JC, Glavina T, Goodstein D, Hadi MZ, Hellsten U, Hildebrand M, Jenkins BD, Jurka J, Kapitonov VV, Kröger N, Lau WWY, Lane TW, Larimer FW, Lippmeier JC, Lucas S, et al.: **The genome of the diatom *Thalassiosira pseudonana*: ecology, evolution, and metabolism.** *Science* 2004, **306**:79–86.

51. Motamayor JC, Mockaitis K, Schmutz J, Haiminen N, Iii DL, Cornejo O, Findley SD, Zheng P, Utro F, Royaert S, Saski C, Jenkins J, Podicheti R, Zhao M, Scheffler BE, Stack JC, Feltus FA, Mustiga GM, Amores F, Phillips W, Marelli JP, May GD, Shapiro H, Ma J, Bustamante CD, Schnell RJ, Main D, Gilbert D, Parida L, Kuhn DN: **The genome sequence of the most widely cultivated cacao type and its use to identify candidate genes regulating pod color.** *Genome Biol* 2013, **14**:r53.

52. Yang R, Jarvis DE, Chen H, Beilstein MA, Grimwood J, Jenkins J, Shu S, Prochnik S, Xin M, Ma C, Schmutz J, Wing RA, Mitchell-Olds T, Schumaker KS, Wang X: **The Reference Genome of the Halophytic Plant *Eutrema salsugineum*.** *Front Plant Sci* 2013, **4**:46.

53. Jaillon O, Aury J-M, Noel B, Policriti A, Clepet C, Casagrande A, Choisne N, Aubourg S, Vitulo N, Jubin C, Vezzi A, Legeai F, Hugueney P, Dasilva C, Horner D, Mica E, Jublot D, Poulain J, Bruyère C, Billault A, Segurens B, Gouyvenoux M, Ugarte E, Cattonaro F, Anthouard V, Vico V, Del Fabbro C, Alaux M, Di Gaspero G, Dumas V, et al.: **The grapevine genome sequence suggests ancestral hexaploidization in major angiosperm phyla**. *Nature* 2007, **449**:463–467.

54. Prochnik SE, Umen J, Nedelcu AM, Hallmann A, Miller SM, Nishii I, Ferris P, Kuo A, Mitros T, Fritz-Laylin LK, Hellsten U, Chapman J, Simakov O, Rensing SA, Terry A, Pangilinan J, Kapitonov V, Jurka J, Salamov A, Shapiro H, Schmutz J, Grimwood J, Lindquist E, Lucas S, Grigoriev IV, Schmitt R, Kirk D, Rokhsar DS: **Genomic analysis of organismal complexity in the multicellular Chlorophyte *Volvox carteri*.** *Science* 2010, **329**:223–226.

55. Schnable PS, Ware D, Fulton RS, Stein JC, Wei F, Pasternak S, Liang C, Zhang J, Fulton L, Graves TA, Minx P, Reily AD, Courtney L, Kruchowski SS, Tomlinson C, Strong C, Delehaunty K, Fronick C, Courtney B, Rock SM, Belter E, Du F, Kim K, Abbott RM, Cotton M, Levy A, Marchetto P, Ochoa K, Jackson SM, Gillam B, et al.: **The B73 maize genome: complexity, diversity, and dynamics.** *Science* 2009, **326**:1112–1115.
