# Supplementary material for: The plant Polycomb repressive complex 1 (PRC1) existed in the ancestor of seed plants and has a complex duplication history
Source: BMC Evol Biol. 2015 Mar 13;15:44. doi: 10.1186/s12862-015-0319-z (PMC4397884; doi:10.1186/s12862-015-0319-z)
Supplement: Additional file 6: — VRN1 gene tree. [file 12862_2015_319_MOESM6_ESM.pdf]

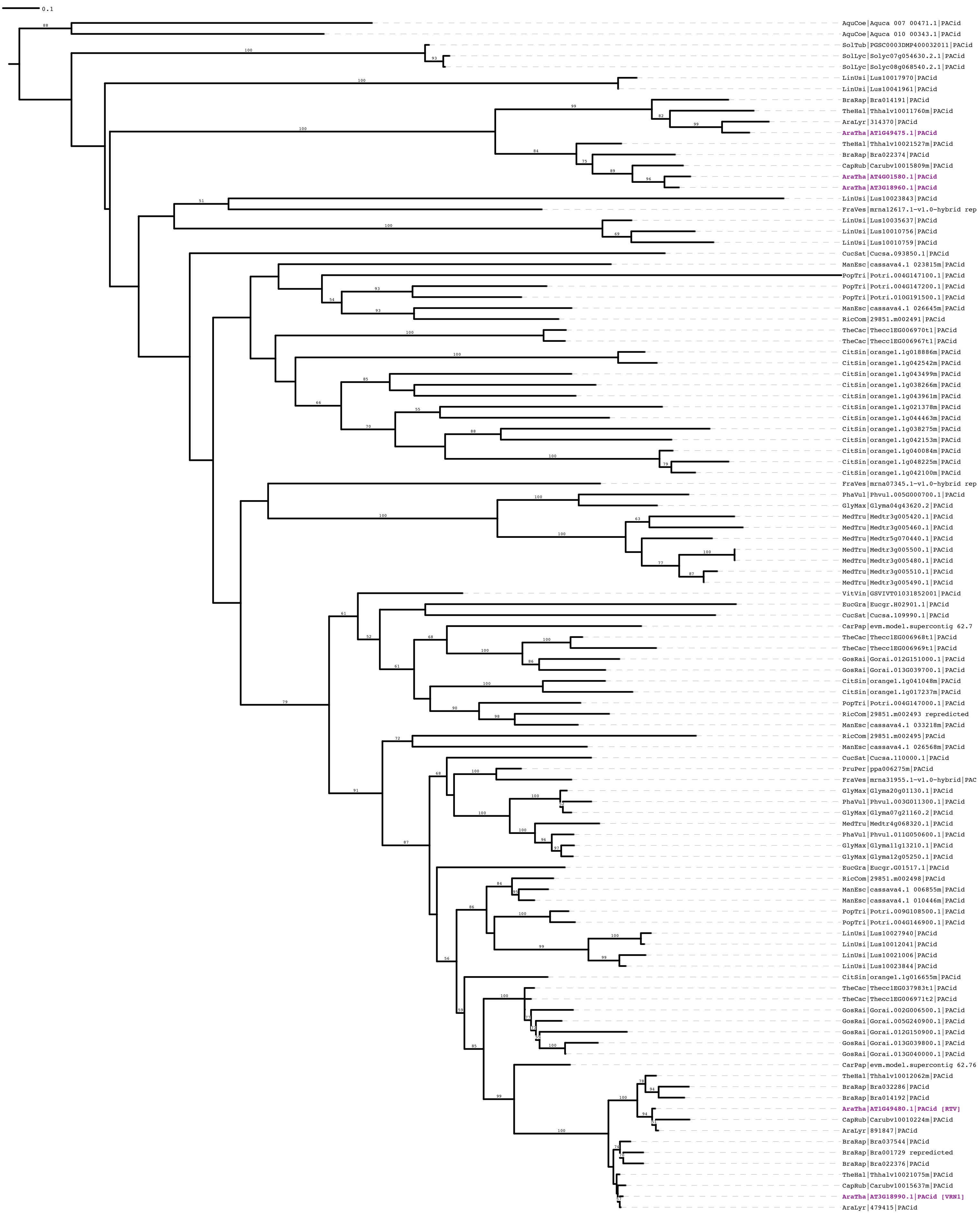

Additional file 6. VRN1 gene tree. Only bootstrap values higher than 50 are shown. *A. thaliana* genes are highlighted in purple.
